# Catalysis of Silver and Bismuth in Various Epoxy Resins

**DOI:** 10.3390/polym16030439

**Published:** 2024-02-05

**Authors:** Hayun Jeong, Keon-Soo Jang

**Affiliations:** Department of Polymer Engineering, School of Chemical and Materials Engineering, The University of Suwon, Hwaseong 18323, Gyeonggi-do, Republic of Korea

**Keywords:** catalysis, metal catalysts, bismuth, copper, epoxy, anhydride, diacid, amine

## Abstract

Epoxy resins find extensive utility across diverse applications owing to their exceptional adhesion capabilities and robust mechanical and thermal characteristics. However, the demanding reaction conditions, including extended reaction times and elevated reaction temperature requirements, pose significant challenges when using epoxy resins, particularly in advanced applications seeking superior material properties. To surmount these limitations, the conventional approach involves incorporating organic catalysts. Within the ambit of this investigation, we explored the catalytic potential of metallic powders, specifically bismuth (Bi) and silver (Ag), in epoxy resins laden with various curing agents, such as diacids, anhydrides, and amines. Metallic powders exhibited efficacious catalytic activity in epoxy–diacid and epoxy–anhydride systems. In contrast, their influence on epoxy–amine systems was rendered negligible, attributed to the absence of requisite carboxylate functional groups. Additionally, the catalytic performance of Bi and Ag are different, with Bi displaying superior efficiency owing to the presence of inherent metal oxide layers on its powder surfaces. Remarkably, the thermal and mechanical properties of uncatalyzed, fully cured epoxy resins closely paralleled those of their catalyzed counterparts. These findings accentuate the potential of Bi and Ag metal catalysts, particularly in epoxy–diacid and epoxy–anhydride systems, spanning a spectrum of epoxy-based applications. In summary, this investigation elucidates the catalytic capabilities of Bi and Ag metal powders, underscoring their ability to enhance the curing rate of epoxy resin systems involving diacids and anhydrides but not amines. This research points toward a promising trajectory for multifarious epoxy-related applications.

## 1. Introduction

Epoxy resins have emerged as a versatile and extensively employed material in various industrial sectors, encompassing electronics, aerospace, automotive, and construction [1,2,3,4,5]. Their utility is grounded in a combination of remarkable qualities, including excellent adhesion, facile formulation and fabrication, cost-effectiveness, lightweight nature, and commendable mechanical and thermal characteristics [6,7,8,9,10]. Nonetheless, these benefits coexist with certain drawbacks, such as brittleness, susceptibility to ultraviolet (UV) degradation, and the prolonged curing process [11,12,13]. These distinctive traits stem from the intricate molecular architecture that incorporates oxirane groups, thus contributing to reactivity and hydroxyl moieties that influence adhesion as a pivotal factor and reactivity as a secondary consideration [14]. This molecular configuration stands as a function of the specific curing agents (hardeners) and epoxy binders in tandem with their ratios. The resultant curing process triggered by these agents leads to the formation of a robust 3D network [15].

Over the preceding decades, extensive efforts have been devoted to expanding the range of properties exhibited by epoxy resin, thus widening their range of application [1,2]. A paramount avenue for manipulating these properties involves the deliberate adjustment of composition through the integration of diverse additives [13,15]. These additives include fillers, reinforcement, plasticizers, elastomers, antifoaming agents, leveling agents, thixotropic agents, thickeners, UV and heat stabilizers, flow control agents, colorants, pigments, adhesion promoters, flame retardants, accelerators, and catalysts. Among these, catalysts play a primary role in governing the kinetics of epoxy resins [15]. Conventionally, the curing of epoxy resins necessitates elevated temperatures and prolonged curing periods, thus leading to complexity, increased energy consumption, and safety considerations [16]. Therefore, catalysts emerge as a potent avenue for addressing these challenges.

Catalysts possess the ability to accelerate the curing reactions of epoxy resins, thus offering the advantages of shortened curing times and reduced temperatures. This leads to more streamlined processing, reduced energy consumption, and enhanced efficiency [17,18,19]. Catalysts primarily influence the curing kinetics while exerting minimal impact on the ultimate properties of the cured epoxy. Diverse catalysts, ranging from highly efficient to less efficient options, find applications in epoxy systems. These include Lewis acids (e.g., boron trifluoride and its complexes), imidazoles (e.g., 1-methyl imidazole), and ureas (e.g., 1,3-dimethyl-3,4,5,6-tetrahydro-2(1H)-pyrimidinone (DMPU)) [13,16,20,21]. Notably, the optimal catalysts vary among different epoxy systems, encompassing epoxy–amine, epoxy–anhydride, and epoxy–diacid subclasses [15,16].

A variety of amine-based curing agents, including aliphatic, alicyclic, aliphatic–aromatic, and aromatic types, are used in epoxy–amine curing systems. While epoxy–aliphatic amine systems can self-cure without catalysts, the use of catalysts can help counteract vitrification and accelerate the reaction. Notably, tertiary amines, exemplified by benzyldimethylamine (BDMA) and triethylamine, serve as catalysts [22,23]. Phenolic compounds can also accelerate the curing reaction of epoxy–amine systems [24,25]. Among the catalysts, boron trifluoride complexes, particularly borontrifluoride monoethylamine (BF_3_·MEA), are the most commonly utilized in epoxy–amine systems [26]. While anhydrides are primarily recognized as hardeners [27], they can also function as accelerators in some formulations of epoxy–amine systems. However, their use may introduce complexities owing to potential reactions among amine, acid, and epoxide groups. For epoxy–anhydride systems, imidazoles (1-methylimidazole, 2-methylimidazole, and 2-ethyl-4-methylimidazole) are extensively acknowledged as effective catalysts [28]. Within the realm of epoxy–anhydride systems, organometallic compounds, such as dibutyltin dilaurate (DBTDL), dibutyltin diacetate (DBTDA), and stannous octoate, play a significant catalytic role by facilitating the formation of carboxylate intermediates that effectively engage with epoxy moieties [29,30,31,32].

However, an intriguing gap remains in that metals or metal-based compounds beyond the tin-based organometallic compounds have not been extensively explored. To address this gap, this study delves into the catalytic capabilities of metals such as Cu and Bi in epoxy resins, shedding light on their nuanced influence on curing processes and property improvement. This investigation encompasses various parameters, including peak reaction temperature, rheology, and thermal and mechanical properties. Understanding the significance of metal catalysts in epoxy systems holds promise for unlocking further potential within epoxy resins, ultimately paving the way for advanced materials characterized by unparalleled versatility.

## 2. Experimental Section

### 2.1. Materials

The epoxy resin used in this study, known as diglycidyl ether of bisphenol A (DGEBA), has an equivalent weight of 187 g/eq. and was supplied by Kukdo Chemicals Co. (Seoul, Republic of Korea). The average repeating unit (n) of the epoxy monomer is 0.12, determined based on the molecular weight of the DGEBA binder (340 + 284 n g/mol).

The curing agents used in the curing process are as follows. Acid compounds include adipic acid (AA, Hojeonable Co., Daejeon, Republic of Korea) and L-glutamic acid (GA, BNOChem Co., Cheongju, Republic of Korea). Anhydride compounds include maleic anhydride (MAn, Tokyo Chemical Industry Co., Tokyo, Japan) and acetic anhydride (AAn, Tokyo Chemical Industry Co., Tokyo, Japan). Amine compounds include bis(4-aminophenyl) sulfone (DDS, SHChemical Co., Cheongju, Republic of Korea) and diamino polypropylene glycol; poly(propylene glycol) bis(2-aminopropyl ether) (D230, MW: 230 g/mol, Kukdo Chemicals Co., Seoul, Republic of Korea).

Metal catalysts used in this study were silver powder (99.0%, ca. 4.4 μm, Kojima Chemicals Co., Saitama, Japan) and bismuth powder (99%, ca. 7.0 μm, melting point: 271 °C, SkySpring Nanomaterials, Inc., Houston, TX, USA).

**Figure 1:**
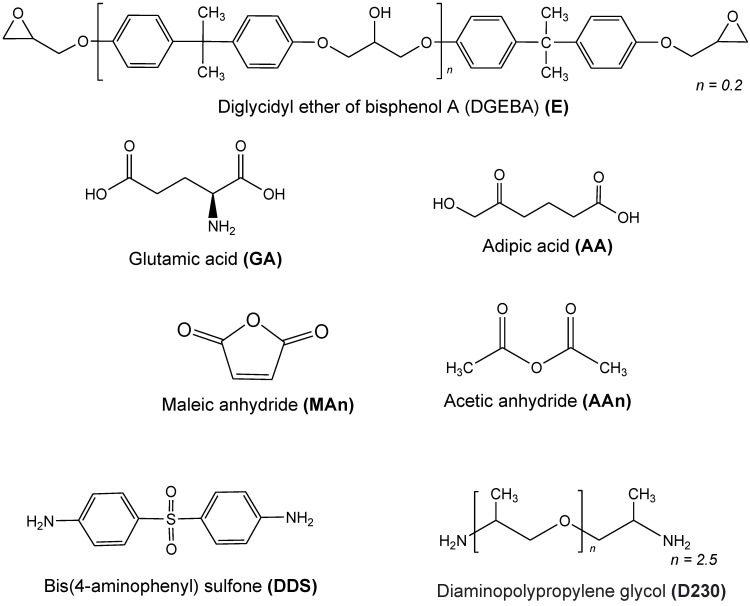
Chemical structures of materials used in this study.

Table 1 presents the names and codes of the chemical substances utilized in this study, and Figure 1 displays their chemical structures.

### 2.2. Compounding Method

The formulation process involved the precise blending of epoxy with each respective curing agent, diacid, anhydride, and amine, in a meticulously balanced 1:1 stoichiometric ratio. This ratio was determined based on the equivalent weight of the epoxy. For instance, 0.39 g of AA (MW: 146 g/mol, EEW: 73 g/eq.), 0.52 g of MAn (MW: 98 g/mol, EEW: 98 g/eq.), and 0.33 g of DDS (MW: 248 g/mol, EEW: 62 g/eq.) were judiciously mixed with 1.00 g of DGEBA, respectively. The ratio between the entire epoxy resin and the corresponding metal powder was linked to volume percentages in this study. The metal powders were added to the mixture in ratios of 0, 1, 5, 10, and 20 vol% at elevated temperatures, where the curing agents reached their molten states. The mixture was stirred for 1–2 min to effectively prevent any unintended propagation of epoxy reactions. Subsequent to this, the mixtures were allowed to cool and then promptly subjected to both differential scanning calorimetry (DSC) and rheological assessments.

### 2.3. Characterization

Differential scanning calorimetry (DSC; DSC25, TA Instruments, New Castle, DE, USA) was utilized to determine the glass transition temperature and the zenith of reaction exothermicity. Approximately 3 mg of epoxy resin—comprising both the epoxy binder and hardener—was hermetically sealed within an aluminum pan and lid assembly. Subsequently, a temperature program was executed in successive phases: an ascent from −60 °C to 300 °C (350 °C for DGEBA (E) homopolymerization and E–MAn heteropolymerization), at a constant heating rate of 10 °C/min, followed by an isothermal dwell at 300 or 350 °C for 1 min. Subsequently, a descent to −60 °C was carried out with a cooling rate of 10 °C/min, followed once again by an isothermal pause of 1 min. The culmination involved a return to 300 °C at the same heating rate of 10 °C/min, held isothermally for another 1 min, and finally, a rapid reduction to 25 °C, facilitated by a cooling rate of 40 °C/min. The glass transition temperatures of the cured samples were measured during the secondary heating scan.

Thermal analysis of epoxy resins with different metal powers and their concentrations was further conducted using thermogravimetric analysis (TGA, Perkins Elmer Co., Waltham, MA, USA) under a N_2_ atmosphere. Samples, averaging 10 mg, were subjected to temperatures ranging from 50 to 500 °C at a rate of 10 °C/min. A nitrogen gas purge was maintained at a flow rate of 50 mL/min.

The evaluation of rheological characteristics was conducted using a rheometer (Discovery Hybrid HR-10 rheometer, TA Instruments, New Castle, DE, USA). Employing a dynamic approach, the viscosities of the various epoxy systems were probed at a resonant frequency of 1 Hz, all under a controlled heating rate of 10 °C/min. The experimental setup featured a sample geometry with a diameter of 8 mm and thickness of 1000 µm. In accordance with the established protocol, the strain and torque were set at 0.1% and 20 µN·m, respectively.

Field emission-scanning electron microscopy (FE-SEM; Apreo, FEI Co., Hillsboro, OR, USA) was utilized to analyze the morphologies of metal-embedded epoxy matrices. The electron beam voltage was 5 kV. The samples were sputter-coated with platinum prior to FE-SEM analysis. Energy-dispersive spectroscopy (EDS) dot mapping was conducted in the EDS mode to observe the dispersion of metal powders in the epoxy matrices by detecting Ag and Bi element distributions.

Mechanical characterization was carried out using a universal testing machine (UTM, DUT-500CM, Daekyung Engineering Co., Bucheon, Gyeonggi-do, Republic of Korea). In line with the experimental design, epoxy resin specimens were prepared with a 1:1 stoichiometric ratio, meticulously injected into silicone molds. These molds were subsequently cured at 150 °C for duration of 12 h within an oven environment. Tensile strength measurements were conducted with a gauge length of 1 mm and controlled displacement velocity of 5 mm/min. The mean mechanical property values were derived from five specimens for each unique sample.

For a comprehensive assessment of moduli, dynamic mechanical analysis (DMA, DMA850, TA Instruments, New Castle, DE, USA) was used. The experimental setup closely mirrored the UTM procedure. Sinusoidal oscillations were applied to the samples, featuring an amplitude of 20 μm and a frequency of 1 Hz. The temperature ranged from 25 °C to 180 °C over time, with a gradual ramping rate of 3 °C/min, to determine the modulus dynamics.

## 3. Results and Discussion

The shift from thermoset (thermosettable) resins to thermoset polymers often involves a noticeable catalytic influence, measured quantifiably by the degree of conversion and its subsequent effect on the glass transition temperature (T_g_). Herein, we examined the catalytic effects introduced by each metallic element (Ag and Bi) in various epoxy matrices. Our investigation had a dual focus on kinetics and mechanical properties. To achieve this comprehensive assessment, we strategically combined analytical techniques, primarily using a combination of DSC and rheological analyses for kinetics and a combination of UTM and DMA methodologies for mechanical properties.

**Figure 2:**
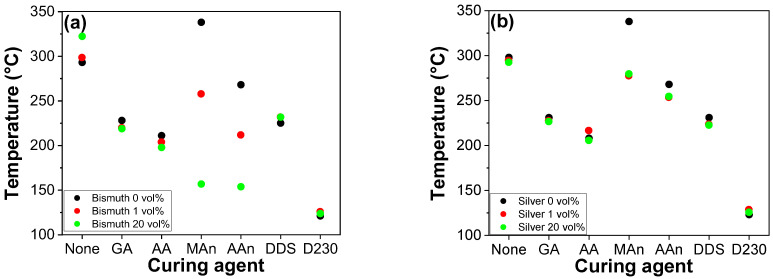
Exothermic reaction peaks exhibited by pristine epoxy binder and hardener-embedded epoxy resins containing Bi (**a**) and Ag (**b**) metal powders with different concentrations of 0, 1, and 20 vol%.

**Figure 3:**
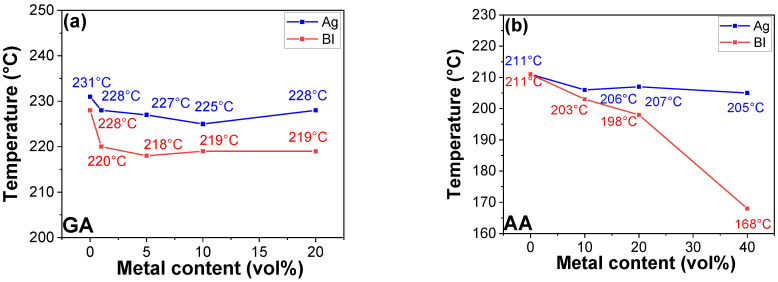

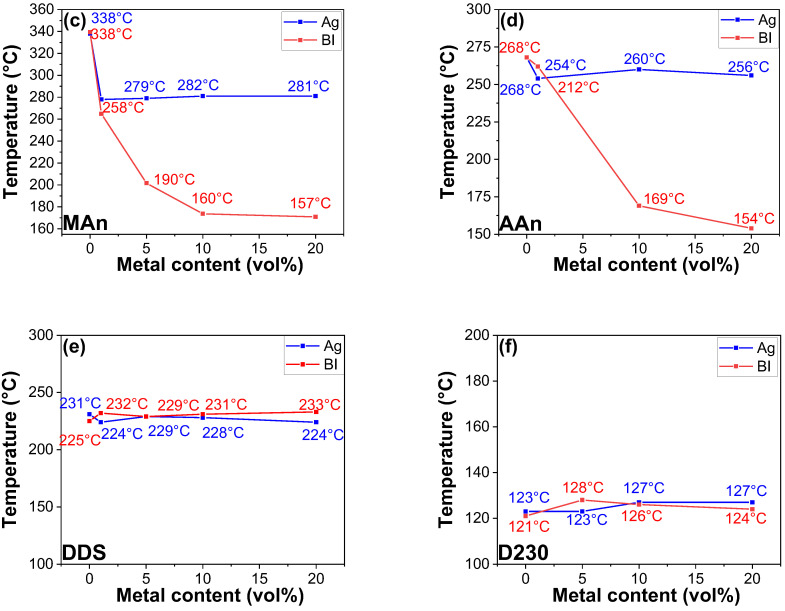
Exothermic reaction peaks for homopolymerization and heteropolymerization of epoxy resins containing Bi and Ag metal powders with different concentrations: (**a**) GA, (**b**) AA, (**c**) MAn, (**d**) AAn, (**e**) DDS, and (**f**) D230.

Figure 2a and Figure 3 depict the discernible effects resulting from the incorporation of Bi in various epoxy resin matrices on curing behaviors, including exothermic reaction peak temperatures. These matrices include no curing agent or different curing agents and varying Bi concentrations, as evidenced by the resulting exothermic reaction peaks (T_peak_). Without a curing agent, no catalytic effect of Bi and Ag was observed, which indicates that the incorporation of Bi and Ag barely influenced the curing behavior of homopolymerization of epoxy resins. It should be noted that the increased exothermic peak of homopolymerization with 20 vol% Bi was derived from the overlap between the endothermic peak (corresponding to the melting point of Bi: ca. 271 °C) and the exothermic peak of homopolymerization. This resulted in a detected T_peak_ higher than the actual value. It is also worth noting that the detected exothermic enthalpy for homopolymerization around 300 °C in Figure 2 was approximately 9 J/g, which is significantly lower than that of heteropolymerization that ranged between 200 and 500 J/g [33,34,35]. Typically, the primary exothermic peak temperature for homopolymerization is detected around 360 °C. However, due to constraints in measurement, the temperature during DSC scans was raised to 350 °C, and as a result, the primary peak was not detected below 350 °C. This implies that the metal powders exerted minimal or no impact on the overall curing behaviors (corresponding to both minor and major exothermic peak temperature) of epoxy homopolymerization in the absence of curing agents. The catalytic effect of Bi was evident across epoxy–diacid, epoxy–amine, and epoxy–anhydride systems, with the former two exhibiting a modest influence. Notably, epoxy–anhydride systems represented a significant reduction in T_peak_, thus signifying high catalytic effects. This catalytic effect was most pronounced in the E-MAn system, where the incorporation of 20 vol% Bi reduced the T_peak_ from its original 338 to 157 °C. The incorporation of 1 vol% Bi into the E-MAn system showed a dramatic decrease in reaction peak temperature from 338 to 258 °C by 80 °C. Notably, catalytic efficacy reached saturation at 10 vol% Bi, thus resulting in a consistent T_peak_ of ca. 160 °C. This trend can be attributed to the inherent differences in reactivity between epoxy–diacid and epoxy–anhydride systems, with epoxy–diacid systems displaying faster reactivity than epoxy–anhydride systems. Although acyclic anhydrides are not typical curing agents for epoxy resins, the acyclic anhydride, AAn, was utilized in this study to examine the effect of cyclization in anhydride structure on reactive behaviors of Bi- and Ag-incorporated epoxy–anhydride systems. It should be noted that cyclic anhydrides, like MAn, are commonly used as curing agents because, in the case of acyclic anhydride, the acyclic molecules undergo cleavage upon reaction with hydroxyl moieties. This disrupts the formation of a robust 3D network structure, leading to poor mechanical and thermal properties.

Conducting similar investigations involving silver (Ag) introduces a distinct dimension to the catalytic discussion in epoxy–amine and epoxy–diacid systems, as evidenced by Figure 2b and Figure 3c,d. By contrast, the addition of a minimal Ag loading (1%) to the epoxy–anhydride systems, namely E-MAn and E-AAn, resulted in depression of T_peak_, from 338 to 279 °C and from 268 to 254 °C, respectively. Overall, the catalytic effects demonstrated by Bi and Ag within the epoxy–anhydride systems were notably pronounced, with Bi exhibiting superior catalytic efficacy compared with Ag. Metal-based compounds are believed to catalyze reactions through the formation of hybrid metal–carboxylate complex structures, analogous to compounds such as dibutyltin dilaurate (DBTDL), dibutyltin diacetate (DBTDA), and stannous octoate. The susceptibility of anhydrides to ambient water and the hydroxyl moieties within epoxy binders lead to the formation of ester and carboxylic acid groups. The generated carboxylic acids derived from curing agents and pre-existing acidic groups inherent in diacid curing agents establish interactions with metal surfaces. Bi, with its metal oxide layer on the surface, can be affected by acidic moieties, thereby producing metal–carboxylate complexes. The generated chemical structure is analogous to organometallic compounds, such as DBTDL, DBTDA, and stannous octoate. They serve as catalysts by facilitating the formation of carboxylate intermediates that accelerate the reactions with epoxy groups [29,30,31,32]. However, in the epoxy–amine systems, regardless of the type of metal, no catalytic effects were observed, likely because of the absence of carboxylate groups.

**Figure 4:**
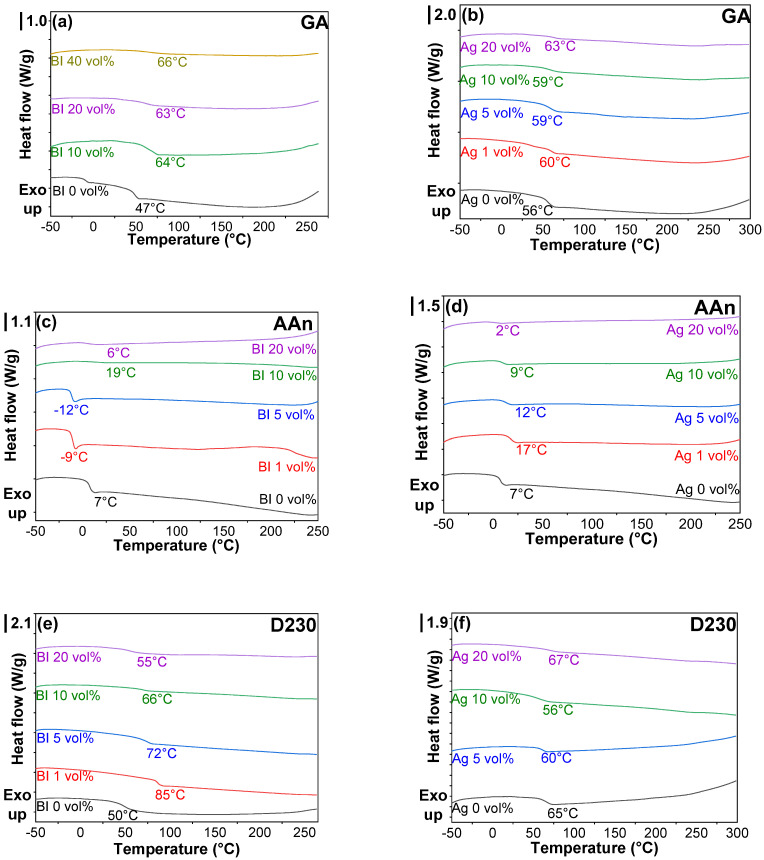
DSC scans of completely cured epoxy resins containing Bi (**a**,**c**,**e**) and Ag (**b**,**d**,**f**) metal powders with different metal concentrations: (**a**,**b**) GA, (**c**,**d**) AAn, and (**e**,**f**) D230.

In addition to their influence on the kinetics of epoxy resins, the comprehensive exploration of the effects induced by Bi and Ag extended to the domain of thermal and mechanical properties, as illustrated in Figure 4. A pivotal parameter reflecting the essential properties of uncured and cured thermoset resins is T_g_. T_g_ can be meticulously determined through an array of methodologies encompassing DMA, DSC, thermomechanical analysis (TMA), and dielectric analysis (DEA). Among these, DMA stands out as a precise method for measuring the T_g_ of cured samples, albeit less suited for assessing uncured, liquid-type specimens. Conversely, DSC offers a straightforward and efficient avenue for evaluating T_g_ in both uncured samples and cured samples.

In addition to determining the T_g_ of cured samples, DSC and DMA were utilized to ascertain the exothermic reaction peak of uncured samples and the modulus of cured counterparts, respectively. The incorporation of Bi or Ag into epoxy resins containing each hardener yielded a marginal or negligible elevation in the T_g_ of cured epoxy matrices, as depicted in Figure 4. This insightful observation underscores that the strategic utilization of Bi and Ag has a minimal discernible impact on molecular architecture. The relatively lower T_g_ values of E-AAn systems without and with metal powders were caused by the absence of cyclic structures, which are necessary to achieve robust 3D networks. Thus, AAn is not used for curing systems. However, AAn was utilized to investigate the reaction rate of acyclic anhydride structures in this study.

**Figure 5:**
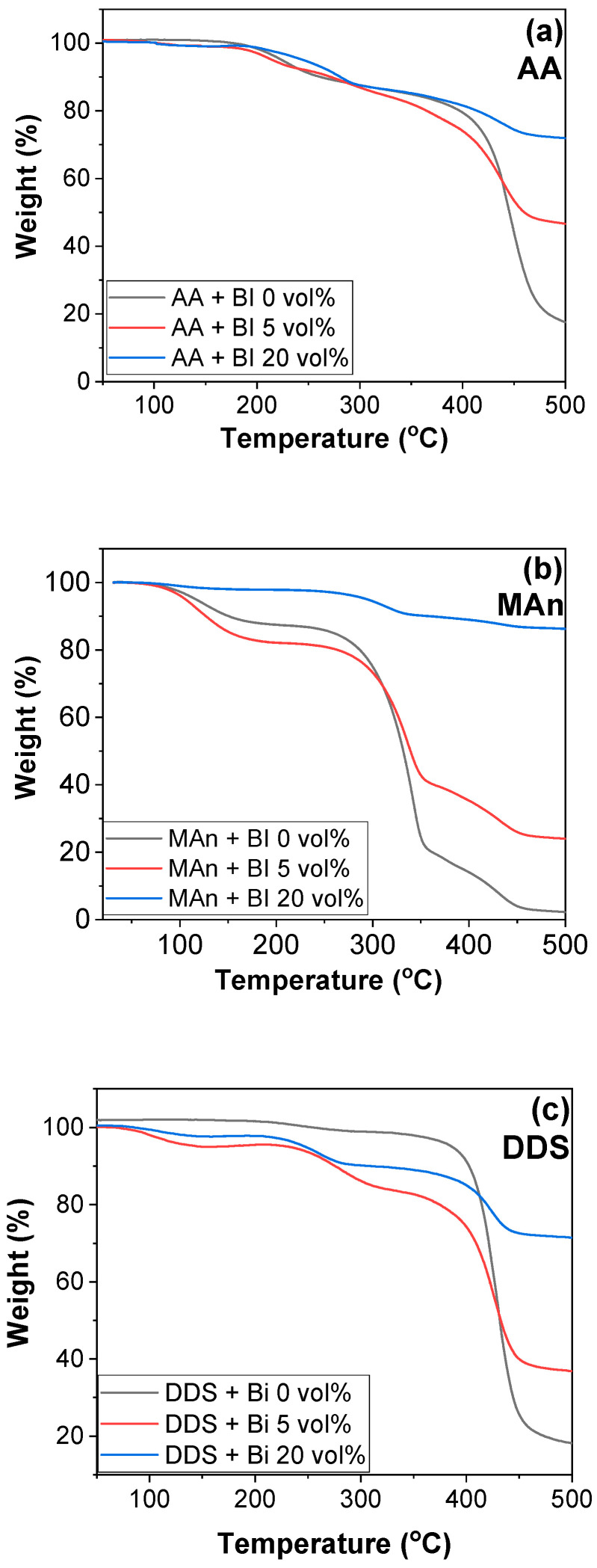
TGA graphs of pristine and Bi-embedded uncured epoxy resins with different curing agents: (**a**) AA, (**b**) MAn, and (**c**) DDS.

In addition to DSC studies, thermal analysis of TGA was examined for uncured samples, as shown in Figure 5. A sharp reduction in weight was observed above 400 °C in most samples. In contrast, the DGEBA-MAn system showed a sharp reduction above 300 °C. However, the least degradation of chemicals was observed in E-MAn with 20 vol% Bi, owing to the chemical reactions catalyzed by Bi, compared to other epoxy systems with 20 vol% Bi. Figures S1a–f and S2a–f show the TGA and dTG (first derivative of TGA) graphs of cured samples, respectively. The cured metal-incorporated samples showed high thermal stabilities compared to the pristine cured samples without metals. The T_d,onset_ and T_d,max_ values were listed based on Figure S2a–f, as shown in Table 2. Similarly, the T_d,onset_ and T_d,max_ values of most cured samples were increased by the incorporation of Bi and Ag into the epoxy matrices. The weight of uncured and cured (150 °C) E-MA with different Ag and Bi loadings was investigated, as shown in Table S1. Any component in samples was barely evaporated during curing, indicating that its composition was maintained until the reaction ended.

**Figure 6:**
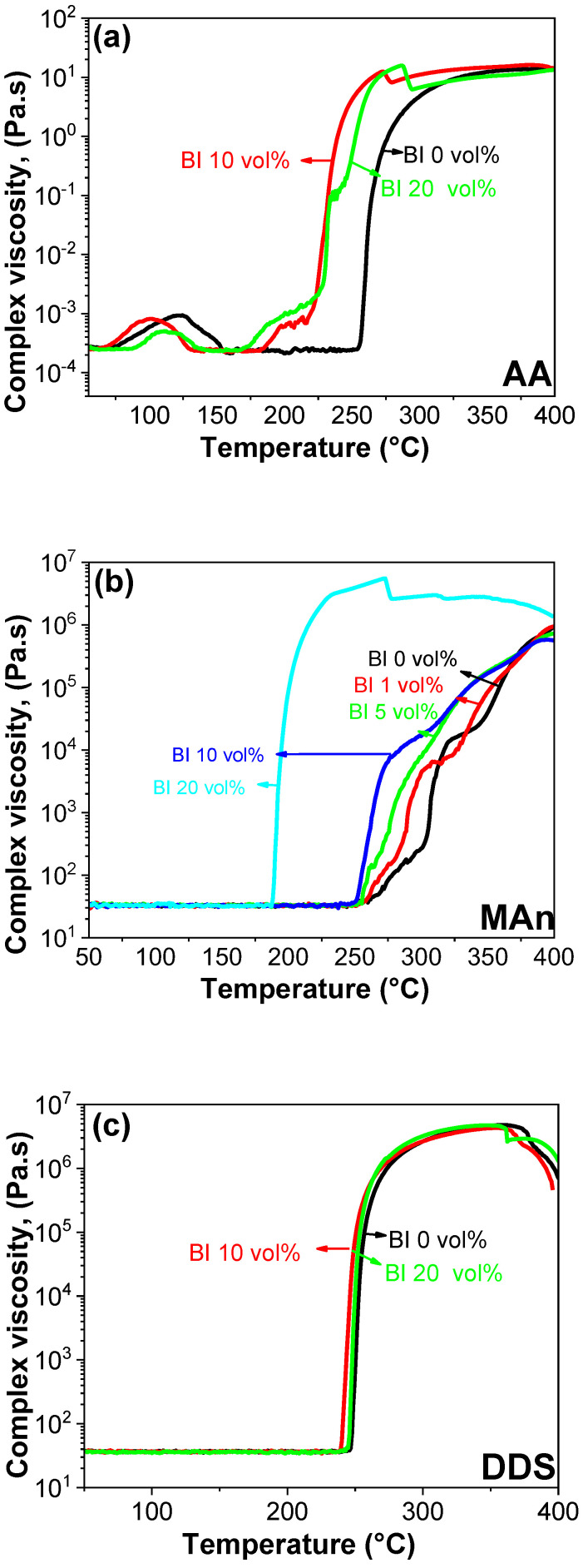
Complex viscosity of pristine and Bi-embedded epoxy resins with different curing agents: (**a**) AA, (**b**) MAn, and (**c**) DDS.

To delve into the intricate realm of rheological properties, which are intricately linked to curing behaviors and reactivity, the complex viscosities of both unaltered and Bi-incorporated epoxy resins hosting distinct curing agents were investigated. Figure 6 presents a cohesive visual representation, with E-AA, E-MAn, and E-DDS serving as representatives for analysis. Consistent with the findings from the DSC assessments, the initiation and completion points of enhanced complex viscosity shifted to lower temperatures with increasing Bi concentration, especially in the epoxy–diacid and epoxy–anhydride systems, as conspicuously portrayed in Figure 6a,b. Notably, the 20 vol% Bi-incorporated E-MAn system exhibited the most significant curve shift towards lower temperatures, indicating a marked reduction in the curing rate. This observation aligns well with the DSC results and was caused by the formation of hybrid metal–carboxylate complex structures, as discussed in DSC results. By contrast, the rheological properties of epoxy–amine systems remained unaffected by the presence or absence of Bi metal powders, emphasizing the conspicuous lack of catalytic effect, a conclusion reinforced by the insights provided in Figure 6c. The key to interpreting the acceleration of the curing reaction catalyzed by Bi lies in its Lewis acidity. A fundamental characteristic of Lewis acids is their ability to accept electrons, thus facilitating the catalysis of covalent bond formation between epoxy resins and their respective curing agents. This orchestration operates by catalyzing the formation of acid-catalyzed ester or anhydride bonds in the case of epoxy–diacid and epoxy–anhydride systems. The presence of Bi accelerated this reaction by providing a Lewis acid site for the acid to react with. In the case of epoxy–amine systems, the curing reaction was initiated by the formation of an acid-catalyzed amine bond. However, Bi lacks a strong Lewis acidity, thereby resulting in minimal catalytic activity in this reaction.

**Figure 7:**
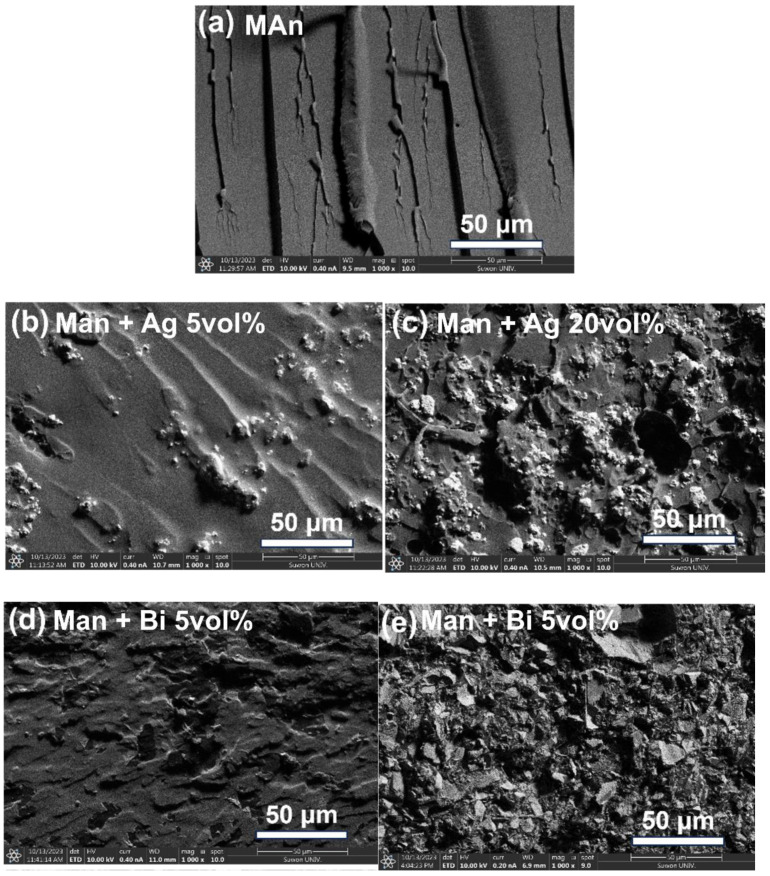
SEM images of fractured surfaces of pristine and Bi-embedded epoxy–anhydride (E-MAn) systems: (**a**) without metal, (**b**) 5 vol% Ag, (**c**) 20 vol% Ag, (**d**) 5 vol% Bi, and (**e**) 20 vol% Bi.

**Figure 8:**
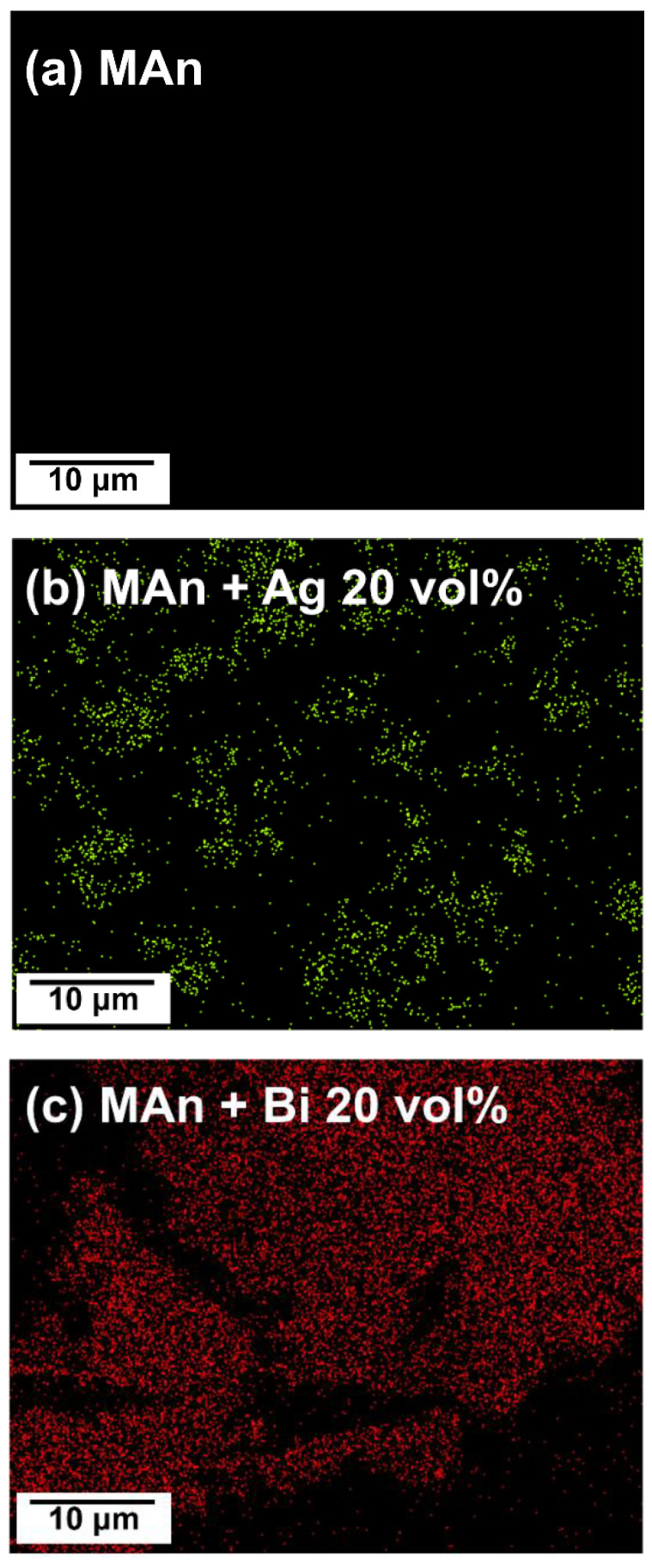
SEM-EDS images of fractured surfaces of pristine and Bi-embedded epoxy–anhydride (E-MAn) systems: (**a**) without metal, (**b**) 20 vol% Ag, and (**c**) 20 vol% Bi.

Morphological analysis of fractured surfaces of both pristine and Ag- or Bi-incorporated E-MA systems was conducted, as depicted in Figure 7 and Figure 8. The curing agents exhibited no agglomeration and did not phase-separate from the epoxy matrix, representing a uniform mixture, as shown in Figure 7a. Nonetheless, the distribution of each metal powder within the epoxy resin matrix was inconsistent across samples, likely owing to the large particle size of the metal powders. The distribution of the metals in other epoxy matrices was also inconsistent, as shown in Figures S3–S10. Enhancing the dispersion of metal powders within the matrix can be achieved through the surface treatment of metal powders, tailoring the viscosity of epoxy resin, and optimizing compounding methods. This can potentially augment the catalysis. In this study, considerable catalytic activity was noted in several systems, even without auxiliary methods, such as surface treatments. It should be noted that the samples containing AAn were unavailable for observing the SEM images owing to the incomplete curing.

**Figure 9:**
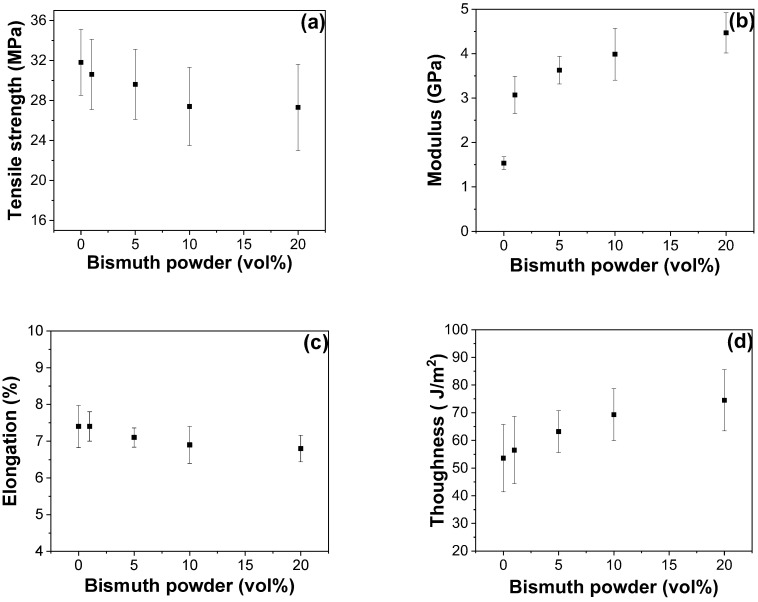
Mechanical properties of pristine and Bi-embedded epoxy–anhydride (E-MAn) systems: (**a**) tensile strength, (**b**) modulus, (**c**) elongation at break, and (**d**) toughness.

The mechanical properties of completely cured epoxy samples, both with and without Bi, were investigated using UTM [36,37,38]. The mechanical test results of Bi-embedded epoxy–anhydride containing MAn as a representative are displayed in Figure 9. The tensile strength and elongation at the break of the cured Bi-embedded epoxy–anhydride (E-MAn) remained largely unchanged with varying Bi concentrations. However, its tensile modulus slightly increased with increasing Bi loading due to the increased elastic behavior (solid-like) caused by the incorporation of metal filler. The toughness also slightly increased due to the elevated modulus. Toughness is derived from the integrated area in a stress–strain curve, inherently linking it to parameters such as strength, modulus, and elongation at break. As depicted in Figure 8, whereas the incorporation of metal powders into the polymer matrix had a minimal impact on both strength and elongation at break, it increased the modulus, thereby increasing the toughness. The incorporation of Bi into the epoxy–anhydride system catalyzed the reaction yet had only a minor influence on the mechanical properties of the final cured sample. This outcome differs from many organic catalyst-triggered chemical reactions because of the presence of metal fillers in this study. The metal fillers can serve as stress concentrators, which may lead to premature material failure. However, they can also increase the toughness of the material by creating a more ductile network. The net effect of these two opposing factors was that the mechanical properties of the cured sample were only slightly affected by the presence of Bi.

**Figure 10:**
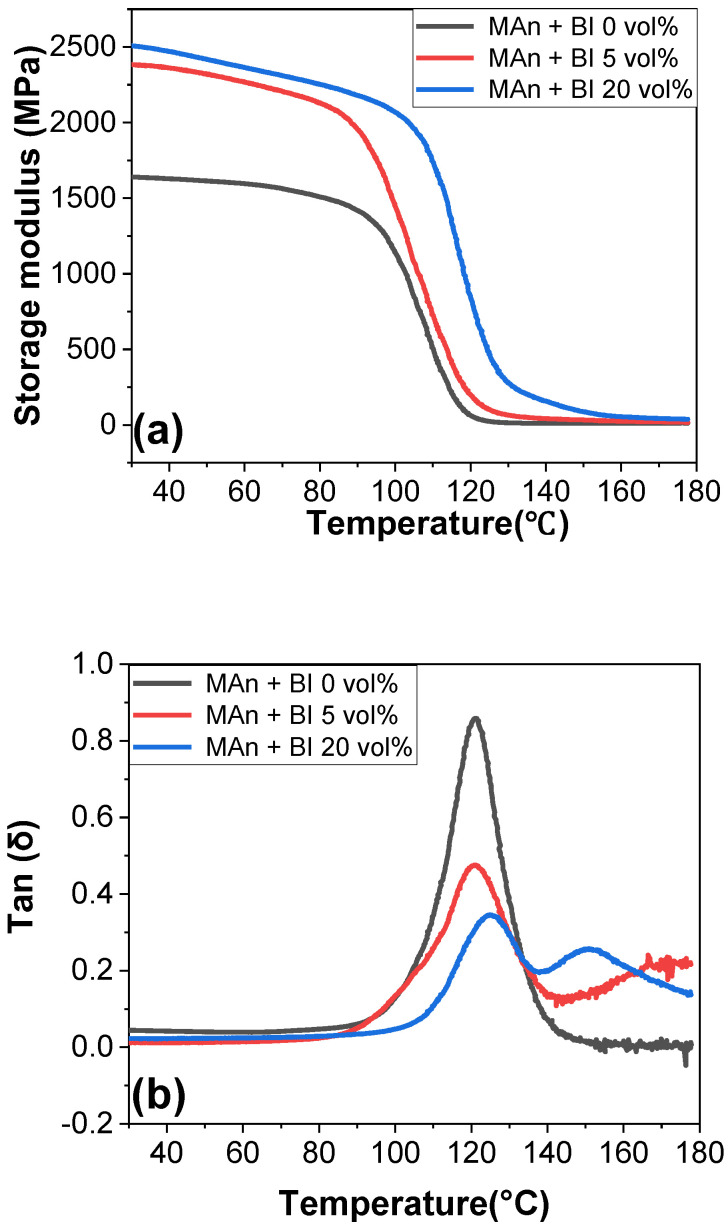
DMA results of E-MAn with different Bi loadings: (**a**) storage modulus; (**b**) Tan δ.

Lastly, DMA was utilized to validate the mechanical and thermal properties of the fully cured epoxy resin infused with Bi and Ag, as shown in Figure 10, Figures S11 and S12. The resulting insights affirmed the earlier observations and established a cohesive framework for the observed phenomena. In alignment with the findings related to tensile modulus, the storage modulus of the sample increased, whereas the tan δ decreased with an increase in Bi concentration. Bi particles can function as filler particles, essentially contributing to the increased material stiffness. This heightened stiffness can lead to an augmentation in the storage modulus. However, in the system of DGEBA-DDS, the incorporation of Ag into the matrix rarely influenced the storage modulus because Ag might impede the curing caused by poor dispersion, and the catalytic effect of Ag was not achieved despite the filler effect. Tan δ is typically small for materials with liquid-like viscous behavior. This indicates that the incorporation of solid-like elastic filler into the epoxy resin enhanced the modulus of the cured sample. Typically, the incorporation of inorganic fillers, including metals, into a polymer matrix increases the modulus. This enhancement stems from various factors, such as the inherent stiffness of the fillers, interfacial interactions, morphological changes (e.g., evolution of crystalline structures in semi-crystalline polymers surrounding the filler particles), constraints of chain mobility, as well as the shape, size, orientation, distribution, and volume fraction of the fillers. For the thermoset epoxy composites investigated in this study, the inherent stiffness of the metal and interfacial interactions were the predominant contributors to the enhanced modulus of the composites. The regions populated by metal powders within the matrix exhibited increased resistance to deformation. In addition, strong adhesion between the hydrophilic metals and the likewise hydrophilic epoxy with generated hydroxyl moieties can effectively transfer load from the matrix to the filler. This load transfer mechanism improved the composite’s resistance to deformation. The combination of these two factors increased the modulus. Its T_g_ based on tan δ slightly increased, which corresponded to the DSC results because of the possibility that the uncurable components prior to degradation in the uncatalyzed system can be cured at low temperatures in the catalyzed systems.

## 4. Conclusions

This study comprehensively investigated the catalytic effects of metallic elements (Ag and Bi) on the curing behaviors, kinetics, and mechanical properties of various epoxy matrices with different curing agents. Our approach integrated a variety of analytical techniques, including DSC and rheological analyses for curing behaviors and UTM and DMA for assessing mechanical properties. We observed that the incorporation of metal powders into epoxy–anhydride systems significantly influenced the curing behaviors. Specifically, a remarkable increase in the curing rate was noted, with the E-MAn system containing 20 vol% Bi showing the most significant shift in T_peak_ from 338 to 157 °C and a decrease in reaction peak temperature by 80 °C with 1 vol% Bi. This effect reached saturation at 10 vol% Bi, maintaining T_peak_ around 160 °C. In homopolymerization without hardeners, the metal powders showed minimal to no impact on curing behaviors. This is highlighted by the lower exothermic enthalpy in homopolymerization (approximately 9 J/g) compared to heteropolymerization (200–500 J/g). In epoxy–amine systems, no catalytic effects were observed for either type of metal, likely due to the absence of carboxylate groups. The incorporation of Bi and Ag marginally influenced the T_g_ of cured epoxy matrices. The tensile strength, elongation at break, and modulus of the cured Bi-embedded epoxy–anhydride system were largely unaffected by varying Bi concentrations. However, a slight increase in modulus and toughness was noted, attributed to the addition of metal filler. The complex viscosity studies revealed that Bi incorporation, particularly in epoxy–diacid and epoxy–anhydride systems, led to a notable shift in viscosity behavior, indicative of an increased curing rate. SEM analysis demonstrated that while the curing agents were uniformly distributed within the epoxy matrix, the dispersion of metal powders was inconsistent, likely due to their large size and heaviness. This suggests that methods to enhance dispersion, such as surface treatment of metal powders and optimization of compounding methods, could further improve catalysis. TGA results confirmed the high thermal stability of metal-incorporated samples, compared to pristine samples. The least degradation was observed in E-MAn with 20 vol% Bi, highlighting the catalytic effects of Bi in improving thermal stability. The catalytic action, particularly of Bi, is attributed to the formation of hybrid metal–carboxylate complex structures, facilitating the curing process. In contrast, the absence of such effects in epoxy–amine systems underscores the pivotal role of carboxylate groups in this catalysis. The introduction of Bi and Ag into epoxy matrices not only catalyzed the curing process but also slightly improved the mechanical properties of the final cured product, such as increased modulus and toughness, without significantly altering the T_g_. This study suggests potential avenues for further enhancing the catalytic and curing behaviors of epoxy systems, such as optimizing metal powder dispersion and exploring different metal types and concentrations. This investigation underscores the significant impact of metal additives on the curing behaviors and mechanical properties of epoxy systems, paving the way for more efficient and enhanced material formulations in industrial applications.

## Figures and Tables

**Table 1:** Chemical substances used in this study.

Type	Full Name	Code
Epoxy	Diglycidyl ether of bisphenol A (DGEBA)	E
Diacid	Glutamic acid	GA
	Adipic acid	AA
Anhydride	Maleic anhydride	MAn
	Acetic anhydride	AAn
Amine	Bis(4-aminophenyl) sulfone	DDS
	Diaminopolypropylene glycol; poly (propylene glycol) bis(2-aminopropyl ether)	D230

**Table 2:** T_d,onset_ and T_d,max_ of pristine and metal-embedded cured epoxy resins with different curing agents, based on dTG graph results: (a) AA, (b) GA, (c) MAn, (d) AAn, (e) DDS, and (c) D230.

	Handener	AA	GA	MAn	AAn	DDS	D230
T_d,onset_ (°C)	No metal	301.9	304.3	293.3	200.0	263.2	302.6
	BI 20 vol%	335.5	309.1	306.0	205.1	389.2	334.8
	Ag 20 vol%	340.2	331.5	296.4	209.4	296.3	306.8
T_d,max_ (°C)	No metal	472.9	448.6	444.9	299.0	404.6	405.3
	BI 20 vol%	471.9	412.7	444.4	306.7	425.1	447.1
	Ag 20 vol%	472.3	435.2	444.3	309.1	412.6	428.6

## Data Availability

Data are contained within the article and supplementary materials.

## Supplementary Materials

The following supporting information can be downloaded at https://www.mdpi.com/article/10.3390/polym16030439/s1; Table S1. Mass of uncured and cured (150 °C) DGEBA-MAn with different Ag and Bi loadings; Figure S1: TGA graphs of pristine and metal-embedded cured epoxy resins with different curing agents: (a) AA, (b) GA, (c) MAn, (d) ACA, (e) DDS, and (c) D230; Figure S2. dTG graphs of pristine and metal-embedded cured epoxy resins with different curing agents: (a) AA, (b) GA, (c) MAn, (d) ACA, (e) DDS, and (c) D230; Figure S3. SEM images of fractured surfaces of DGEBA-AA systems with different metals: (a) None, (b) 20 vol% Bi, and (c) 20 vol% Ag; Figure S4. SEM images of fractured surfaces of DGEBA-GA systems with different metals: (a) None, (b) 20 vol% Bi, and (c) 20 vol% Ag; Figure S5. SEM images of fractured surfaces of DGEBA-DDS systems with different metals: (a) None, (b) 20 vol% Bi, and (c) 20 vol% Ag; Figure S6. SEM images of fractured surfaces of DGEBA-D230 systems with different metals: (a) None, (b) 20 vol% Bi, and (c) 20 vol% Ag; Figure S7. SEM-EDS images of fractured surfaces of DGEBA-AA systems with different metals: (a) None, (b) 20 vol% Bi, and (c) 20 vol% Ag; Figure S8. SEM-EDS images of fractured surfaces of DGEBA-GA systems with different metals: (a) None, (b) 20 vol% Bi, and (c) 20 vol% Ag; Figure S9. SEM-EDS images of fractured surfaces of DGEBA-DDS systems with different metals: (a) None, (b) 20 vol% Bi, and (c) 20 vol% Ag; Figure S10. SEM-EDS images of fractured surfaces of DGEBA-D230 systems with different metals: (a) None, (b) 20 vol% Bi, and (c) 20 vol% Ag; Figure S11. Storage moduli of DGEBA-hardener systems with 20 vol% metal and different hardeners, measured by DMA: (a) AA, (b) DDS, and (c) D230; Figure S12. Tan δ of DGEBA-hardener systems with 20 vol% metal and different hardeners, measured by DMA: (a) AA, (b) DDS, and (c) D230.
