# Supplementary material for: Catalysis of Silver and Bismuth in Various Epoxy Resins
Source: Polymers (Basel). 2024 Feb 5;16(3):439. doi: 10.3390/polym16030439 (PMC10857170; doi:10.3390/polym16030439)
Supplement: Supplementary file 1 [file polymers-16-00439-s001.zip › polymers-2814756-supplementary.pdf]

Supplementary information

Catalysis of silver and bismuth in various epoxy resins

*Hayun Jeong and Keon-Soo Jang**

Department of Polymer Engineering, School of Chemical and Materials Engineering, The University of Suwon, Hwaseong, Gyeonggi-do,18323, Republic of Korea

CORRESPONDING AUTHOR FOOTNOTE *To whom correspondence should be addressed. K.-S. Jang: ksjang@suwon.ac.kr

Table S1. Mass of uncured and cured (150 °C) DGEBA-MAn with different Ag and Bi loadings

Curing time	0 min (g)	1 h (g)	24 h (g)
Pristine epoxy	2.359	2.355	2.351
Bi 5 vol%	2.403	2.391	2.388
Bi 20 vol%	2.590	2.512	2.491
Ag 5 vol%	2.421	2.413	2.381
Ag 20 vol%	2.411	2.409	2.406

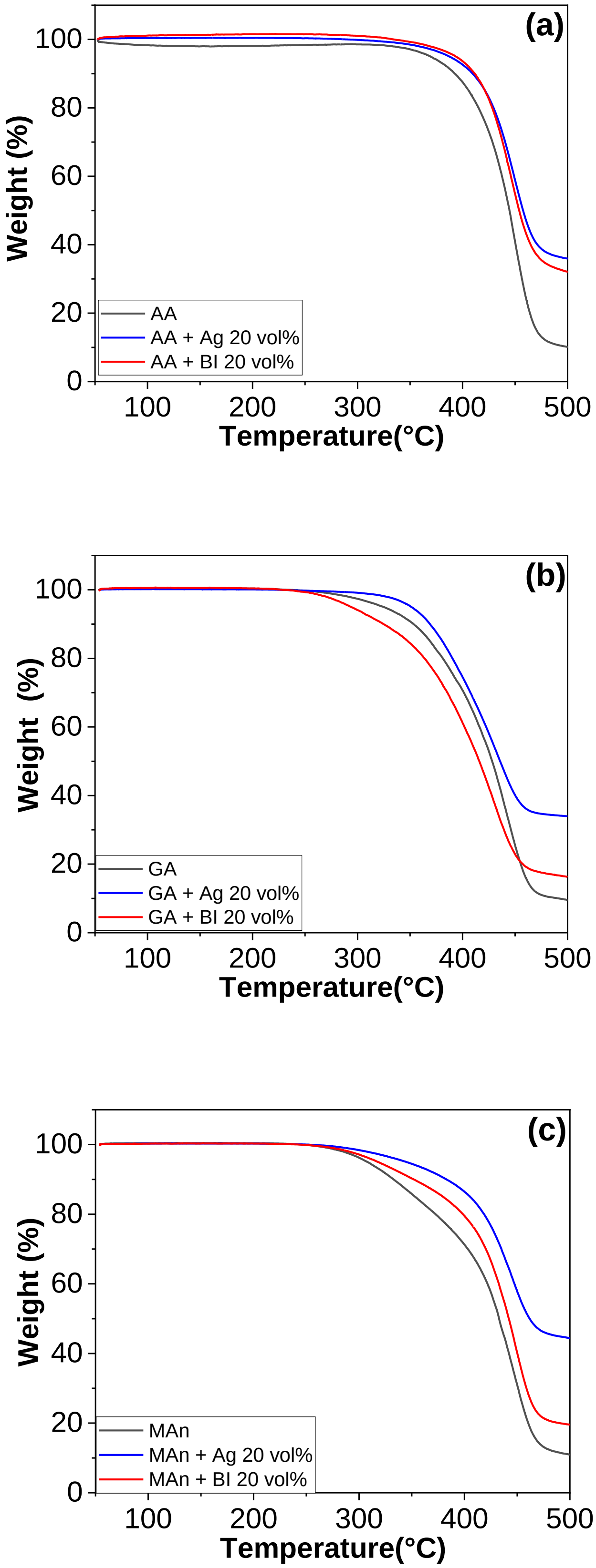
(a)
Weight (%)
Temperature(°C)
AA
AA + Ag 20 vol%
AA + BI 20 vol%
(b)
Weight (%)
Temperature(°C)
GA
GA + Ag 20 vol%
GA + BI 20 vol%
(c)
Weight (%)
Temperature(°C)
MAn
MAn + Ag 20 vol%
MAn + BI 20 vol%

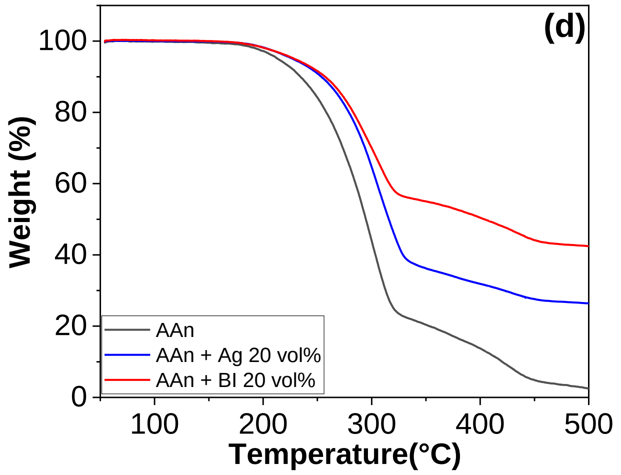

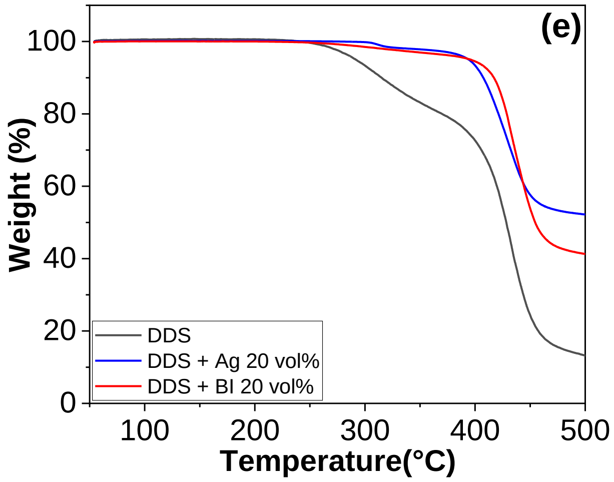

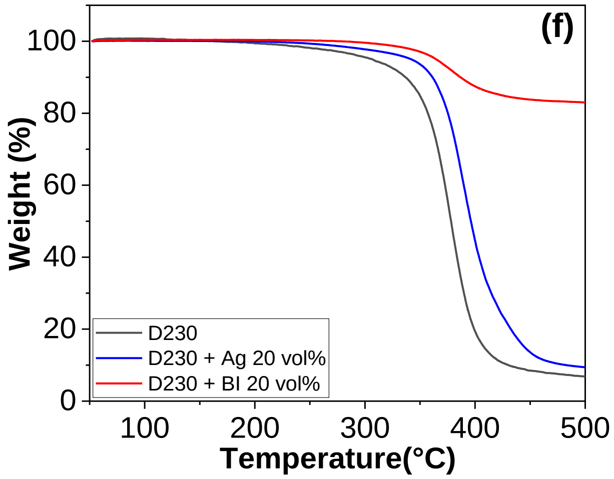

Figure S1. TGA graphs of pristine and metal-embedded cured epoxy resins with different curing agents: (a) AA, (b) GA, (c) MAn, (d) ACA, (e) DDS, and (c) D230.

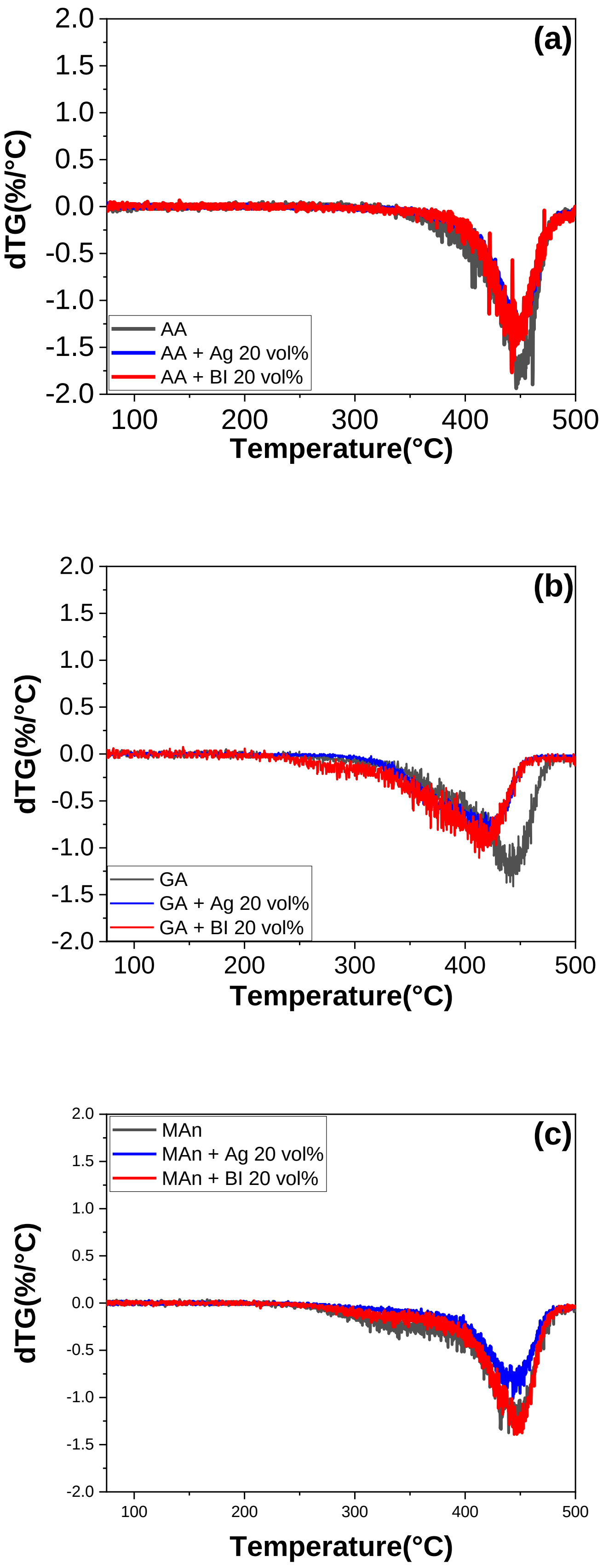
(a)
dTG(%/°C)
Temperature(°C)
AA
AA + Ag 20 vol%
AA + BI 20 vol%
2.0
1.5
1.0
0.5
0.0
-0.5
-1.0
-1.5
-2.0
100
200
300
400
500
(b)
dTG(%/°C)
Temperature(°C)
GA
GA + Ag 20 vol%
GA + BI 20 vol%
2.0
1.5
1.0
0.5
0.0
-0.5
-1.0
-1.5
-2.0
100
200
300
400
500
(c)
dTG(%/°C)
Temperature(°C)
MAn
MAn + Ag 20 vol%
MAn + BI 20 vol%
2.0
1.5
1.0
0.5
0.0
-0.5
-1.0
-1.5
-2.0
100
200
300
400
500

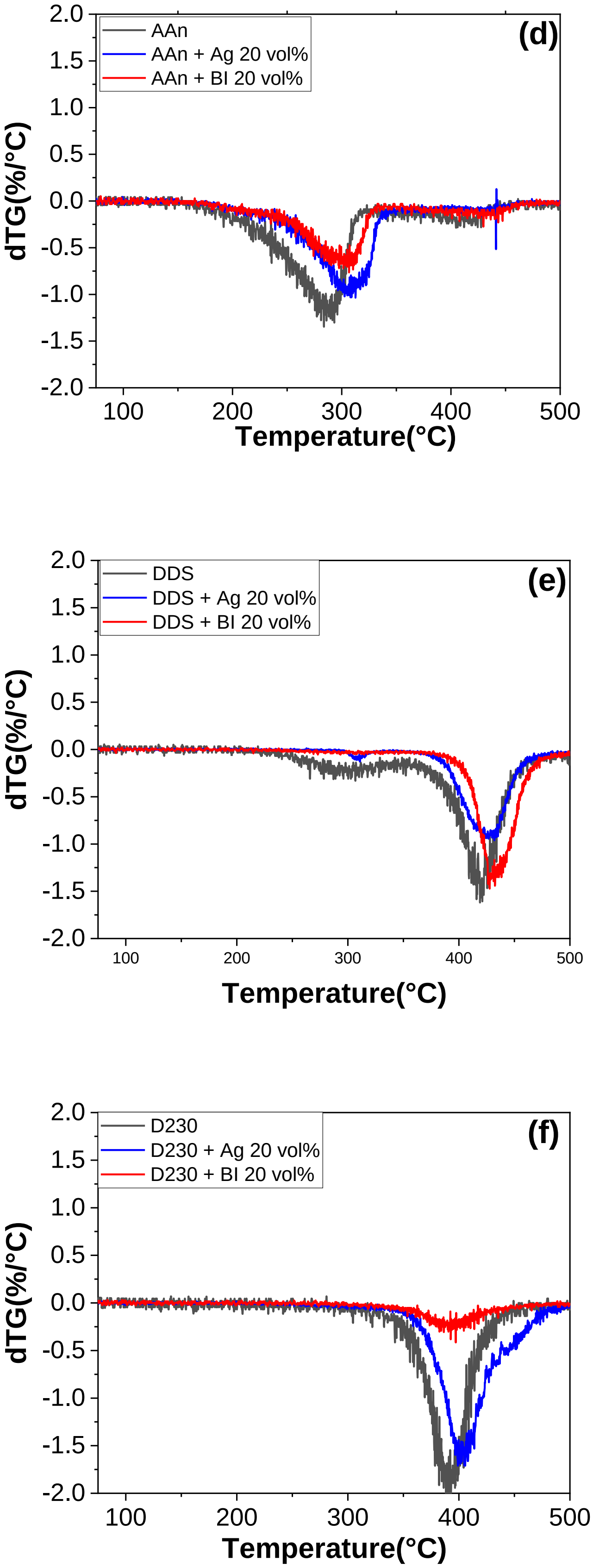

Figure S2. dTG graphs of pristine and metal-embedded cured epoxy resins with different curing agents: (a) AA, (b) GA, (c) MAn, (d) ACA, (e) DDS, and (c) D230.

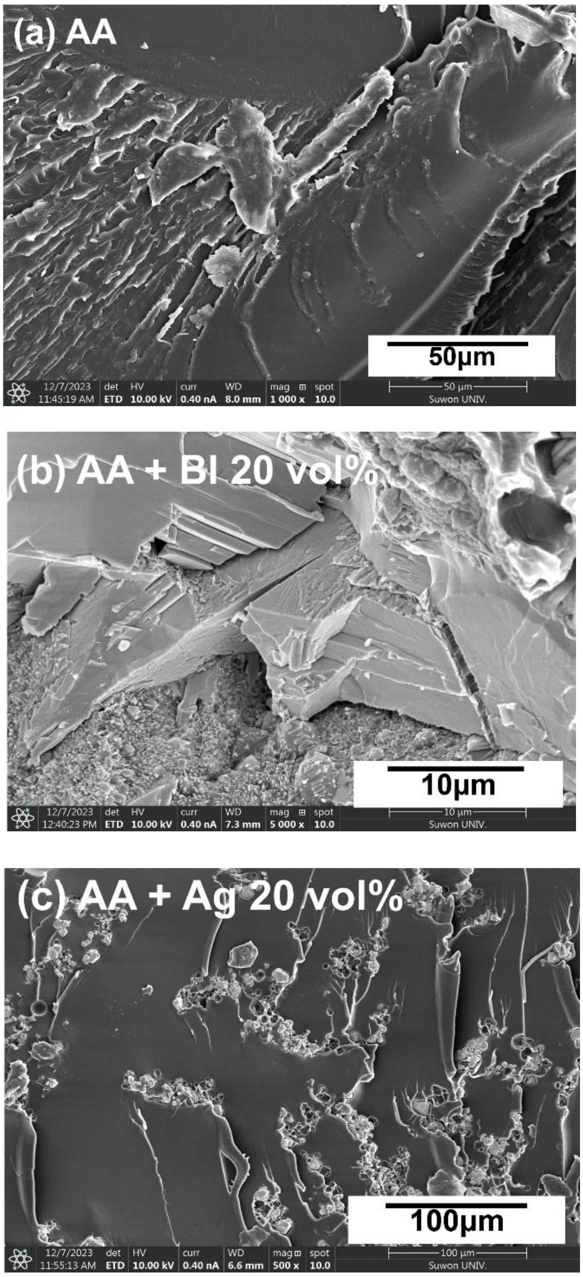

Figure S3. SEM images of fractured surfaces of DGEBA-AA systems with different metals: (a) None, (b) 20 vol% Bi, and (c) 20 vol% Ag.

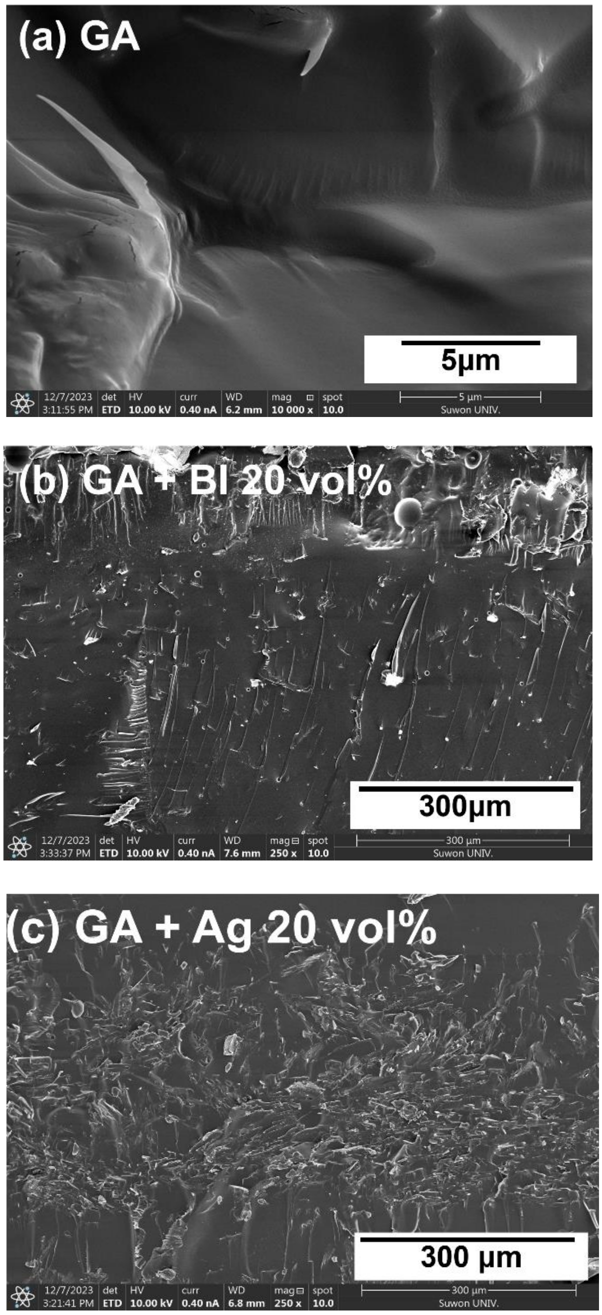

Figure S4. SEM images of fractured surfaces of DGEBA-GA systems with different metals: (a) None, (b) 20 vol% Bi, and (c) 20 vol% Ag.

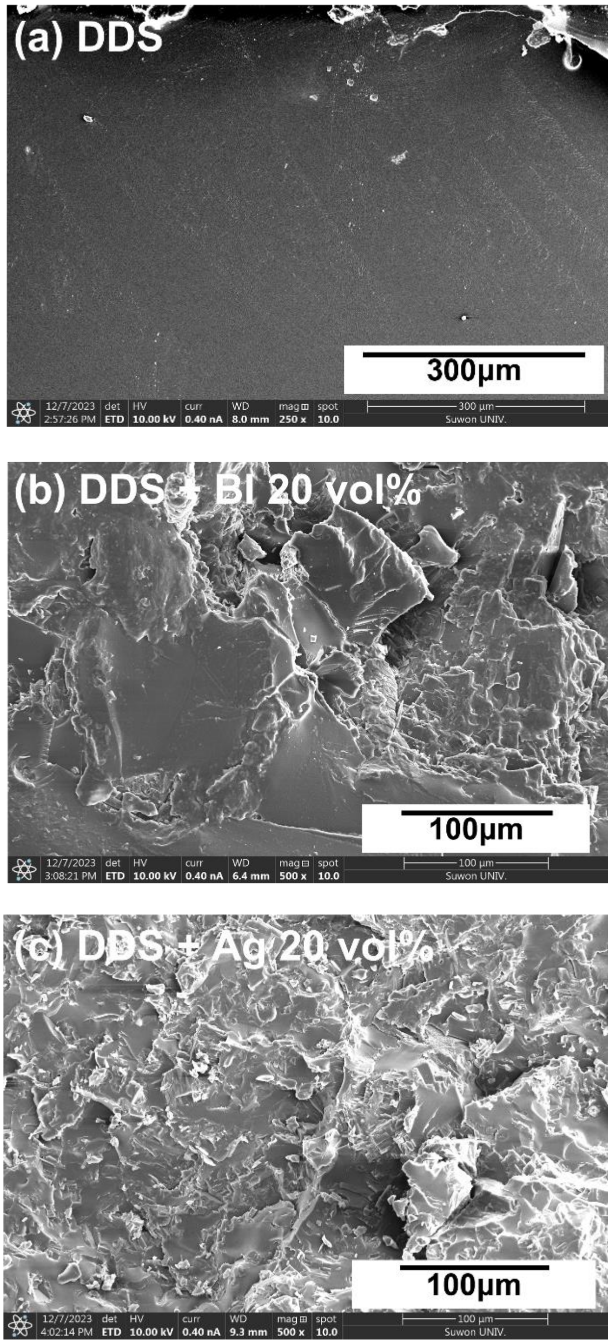

Figure S5. SEM images of fractured surfaces of DGEBA-DDS systems with different metals: (a) None, (b) 20 vol% Bi, and (c) 20 vol% Ag.

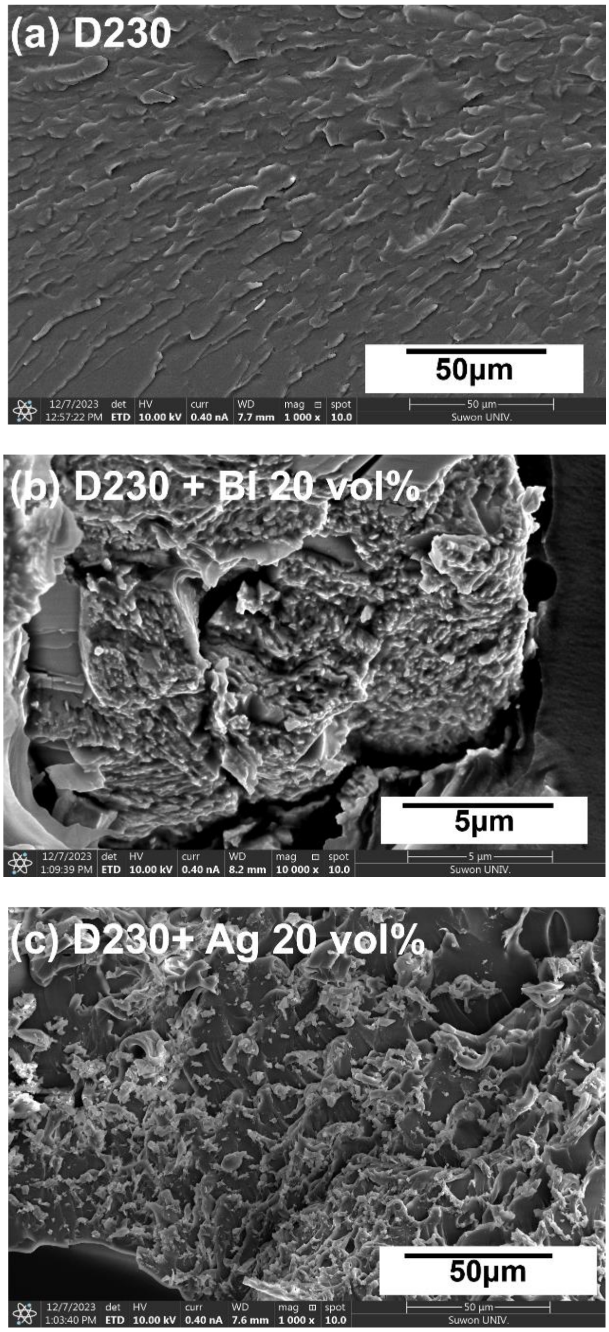

Figure S6. SEM images of fractured surfaces of DGEBA-D230 systems with different metals: (a) None, (b) 20 vol% Bi, and (c) 20 vol% Ag.

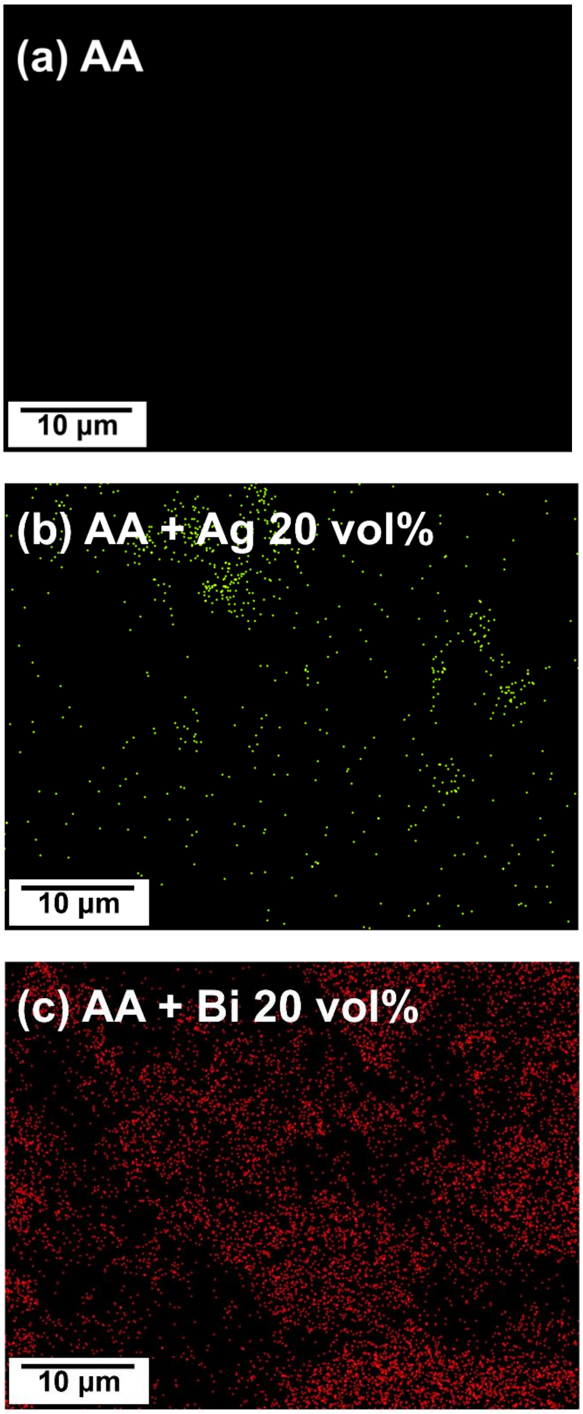

Figure S7. SEM-EDS images of fractured surfaces of DGEBA-AA systems with different metals: (a) None, (b) 20 vol% Bi, and (c) 20 vol% Ag.

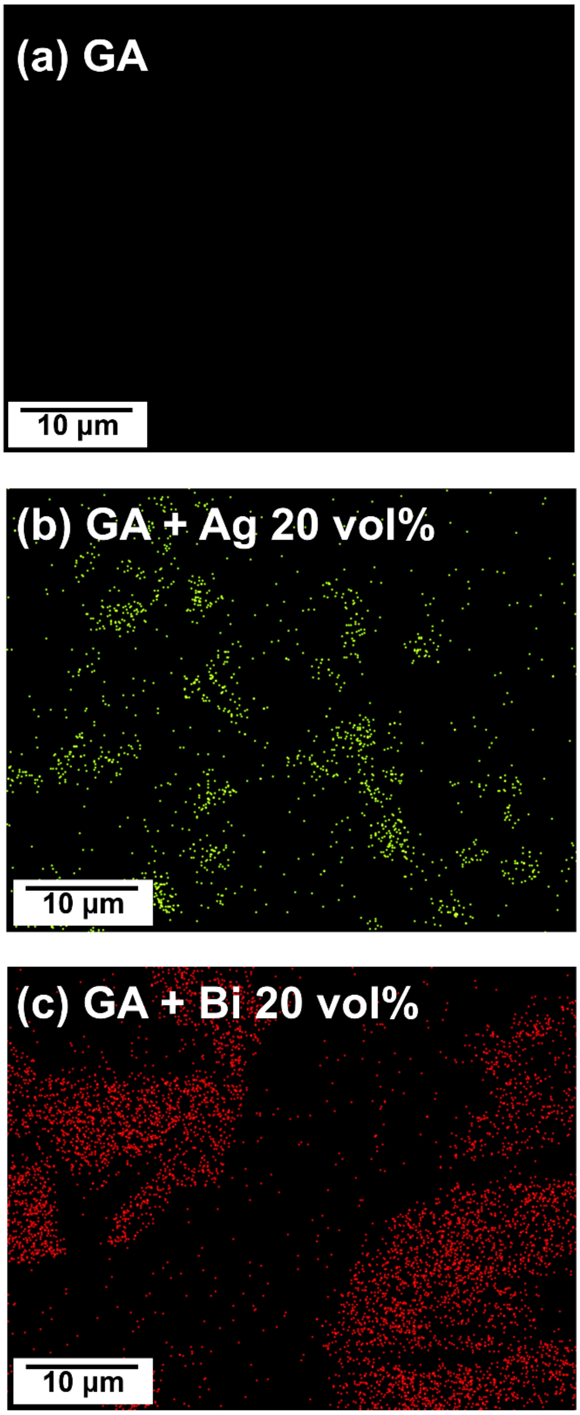

Figure S8. SEM-EDS images of fractured surfaces of DGEBA-GA systems with different metals: (a) None, (b) 20 vol% Bi, and (c) 20 vol% Ag.

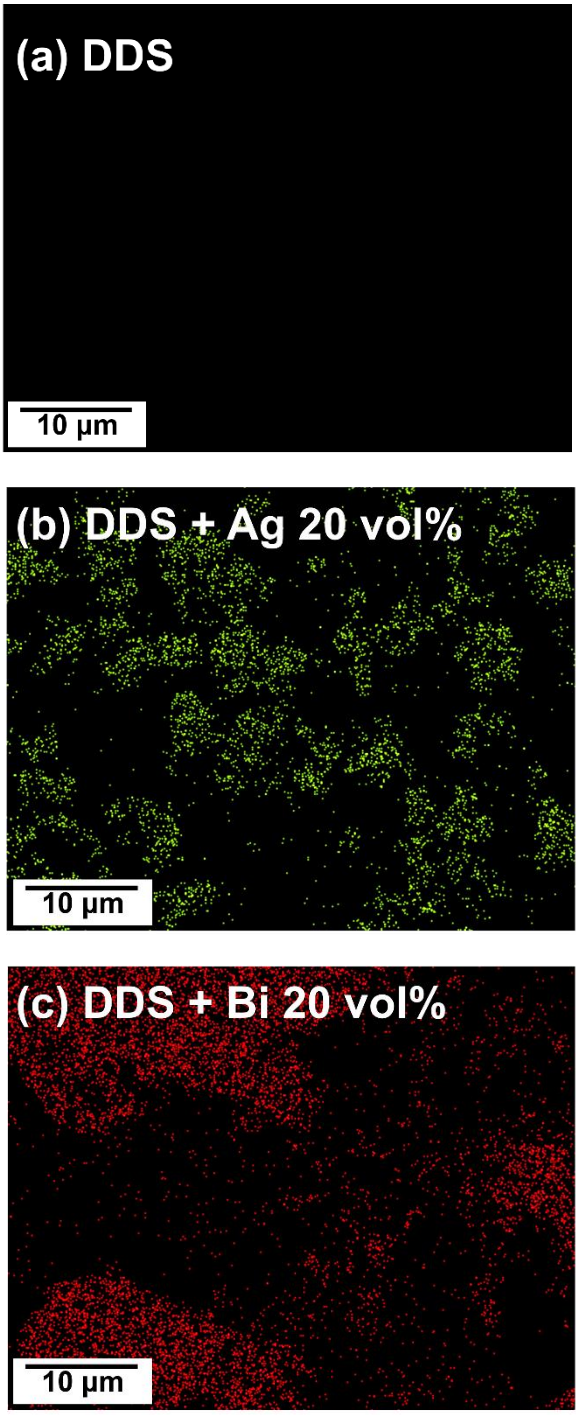

Figure S9. SEM-EDS images of fractured surfaces of DGEBA-DDS systems with different metals: (a) None, (b) 20 vol% Bi, and (c) 20 vol% Ag.

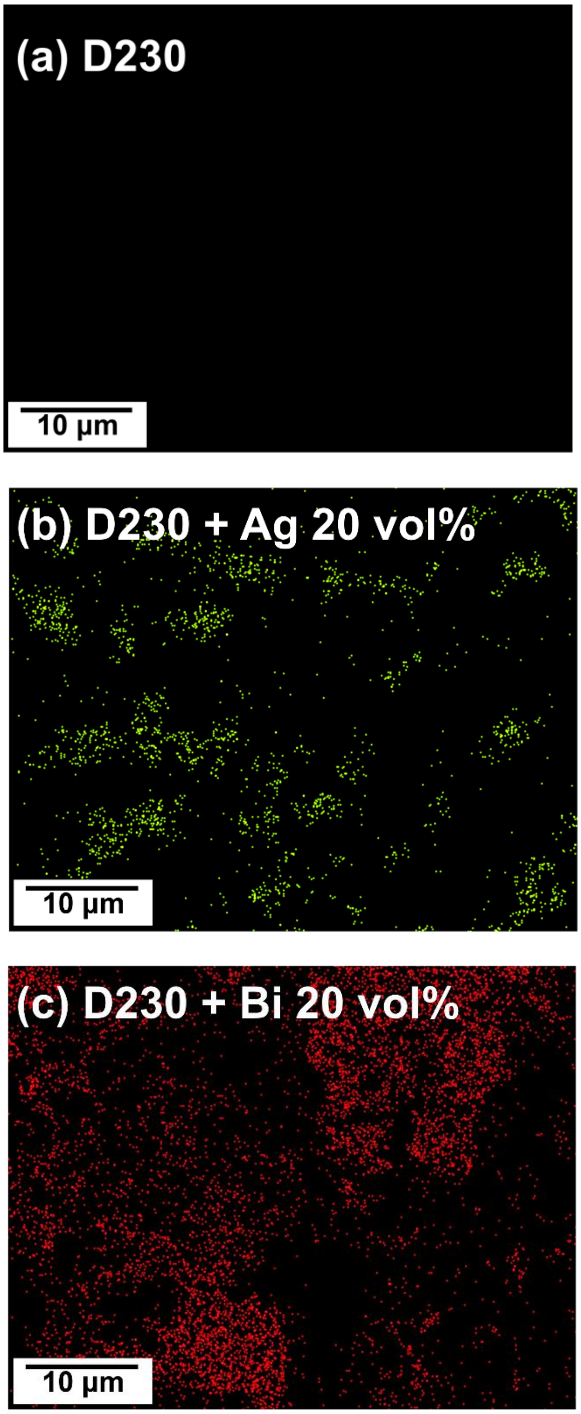

Figure S10. SEM-EDS images of fractured surfaces of DGEBA-D230 systems with different metals: (a) None, (b) 20 vol% Bi, and (c) 20 vol% Ag.

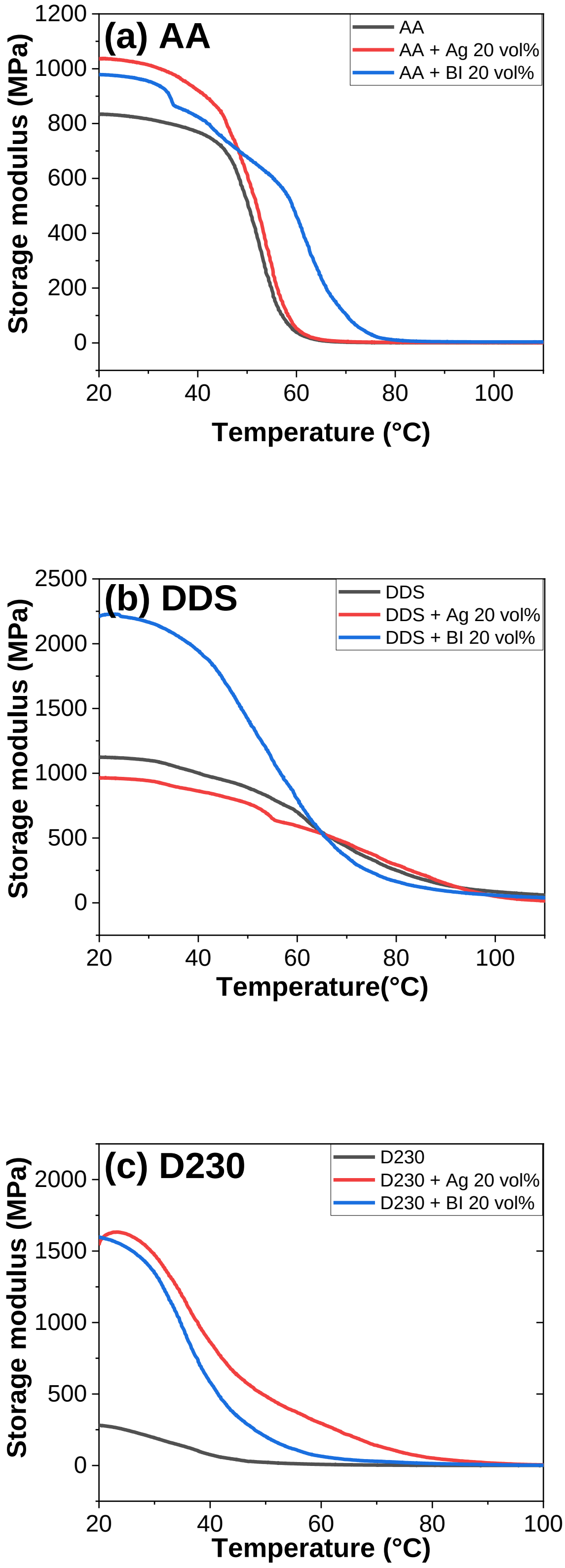

Figure S11. Storage moduli of DGEBA-hardener systems with 20 vol% metal and different hardeners, measured by DMA: (a) AA, (b) DDS, and (c) D230.

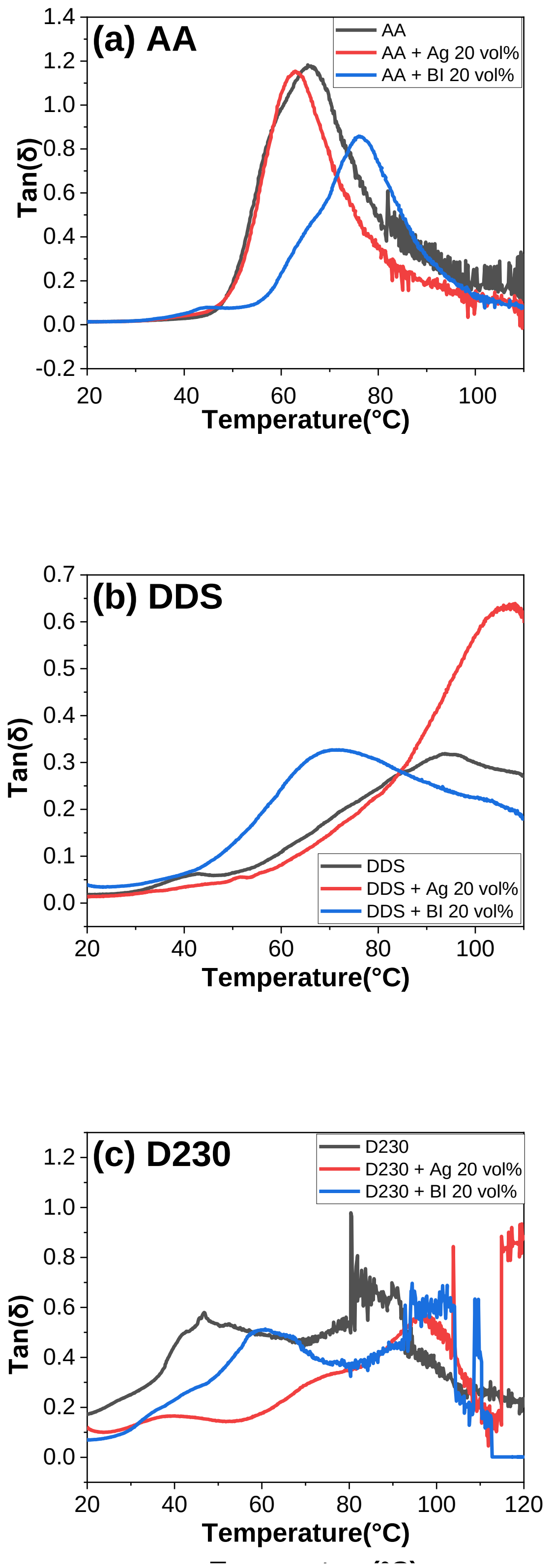

Figure S12. Tan δ of DGEBA-hardener systems with 20 vol% metal and different hardeners, measured by DMA: (a) AA, (b) DDS, and (c) D230.
